# The Role of Neonatal Nurses in Mechanical Ventilation Management

**DOI:** 10.1111/nicc.70150

**Published:** 2025-08-18

**Authors:** Ntombifikile Klaas, Tsholofelo Matlhola

**Affiliations:** ^1^ Department of Nursing Education, School of Therapeutics, Faculty of Health Sciences University of the Witwatersrand Johannesburg South Africa

**Keywords:** mechanical ventilation, neonatal intensive care unit, neonatal nurses, role

## Abstract

**Background:**

Nurses have a critical role in managing mechanical ventilation (MV) in neonatal intensive care units (NICUs). Despite their critical role in day‐to‐day MV management, their role in key decisions, particularly extubating and weaning, remains limited.

**Aim:**

To describe the role of neonatal nurses in MV management in neonatal intensive care units.

**Study Design:**

Descriptive survey design: Data were collected using the *Survey of Mechanical Ventilation and Weaning Roles and Responsibilities* questionnaire. Census sampling was used to select 108 nurses working in NICUs from two university‐affiliated hospitals in Gauteng, South Africa. Descriptive and comparative statistics were applied to analyse the data.

**Results:**

This study achieved an 83.3% response rate, revealing that MV decisions were predominantly collaborative between nurses and doctors. While 90% of nurses were involved in patient evaluation and ventilator adjustments, their role in extubation decisions was limited (45.6%), with doctors making most extubation decisions (54.4%). Oxygen titration was the most frequently managed ventilator setting by neonatal nurses. Nurses' perceived autonomy and influence in MV decision‐making had median scores of 6.0, with higher perceived nursing autonomy significantly linked to independent decision‐making (OR = 1.55; 95% CI = 1.22–1.97; *χ* ^2^(1) = 12.86; *p* < 0.001) and higher influence scores significantly predicting autonomous decisions (OR = 1.86; 95% CI = 1.40–2.47; *χ* ^2^(1) = 18.34; *p* < 0.001). However, only 36% of ICUs had weaning protocols, and ongoing MV education was lacking.

**Conclusion:**

The study underscores the need for enhanced education, structured training and standardised protocols to strengthen nurses' competency, perceived autonomy and confidence in MV management. While nurses actively participate in ventilation‐related decisions, their autonomy remains limited, particularly in extubation decisions.

**Relevance to Clinical Practice:**

Optimising neonatal outcomes requires well‐prepared nurses who can actively and confidently contribute to MV‐related decisions. Enhancing nurses' autonomy through structured education and standardised protocols is crucial for improving neonatal outcomes and promoting safer, more effective care and strengthens collaboration in NICUs.

**Impact Statements**

- What is known about the topic
  ○ Neonatal nurses have a crucial role in the management of mechanical ventilation.
  ○ Mechanical ventilation management protocols are physician‐led and underutilised in ICU settings.
- What this paper adds
  ○ It provides new insights into the role of neonatal nurses in MV management in South Africa, particularly in relation to their level of autonomy and collaborative decision‐making with doctors.
  ○ It highlights the influence of experience and educational background on nurses' involvement in key ventilation decisions, such as weaning and extubation.
  ○ The findings contribute to the growing body of evidence supporting the implementation of structured training programmes and standardised protocols to enhance neonatal nurses' competencies in MV management.

## 1 Introduction and Background

Nurses play an essential role in caring for neonates requiring mechanical ventilation (MV) in neonatal intensive care units (NICUs). Their responsibilities extend beyond basic nursing care, requiring expertise in respiratory physiology, ventilation methods and the clinical observation of neonates [1]. These nurses are tasked with continuous monitoring for signs of respiratory distress, ensuring ventilator settings align with the neonate's needs, collaborating with the multidisciplinary team (MDT) to adjust MV settings, transitioning neonates from invasive to non‐invasive support, facilitating weaning and supporting families emotionally through family‐centred care to alleviate parental anxiety [2, 3].

High‐quality care in the NICU context requires nurses to understand their role in the MDT, particularly in decision‐making related to MV management. However, despite their critical contributions, the role of neonatal nurses in MV management remains underdefined. The existing literature often emphasises physician‐led protocols, leaving the day‐to‐day responsibilities and expertise of neonatal nurses underrepresented [4, 5].

MV is a life‐saving intervention for neonates unable to breathe independently. Early weaning, ideally within the first 3 days, is essential to minimise risks associated with prolonged MV [6]. These risks include ventilator‐induced lung injury (VILI), ventilator‐associated pneumonia (VAP), volutrauma, atelectasis and haemodynamic impairment [7, 8]. Weaning involves gradually reducing respiratory support until the neonate can breathe independently [9].

Weaning from MV is a complex process that requires accurate assessment of the neonate's readiness, safe reduction of ventilator support and timely extubation. Neonates' narrow physiological margins for oxygenation and ventilation make weaning particularly challenging [10, 11]. Neonatal nurses play a critical role in this process by monitoring for signs of respiratory instability, interpreting arterial blood gas results and adjusting ventilator settings as needed [12, 13]. Their ability to recognise readiness for weaning promptly and initiate appropriate interventions is pivotal in reducing the duration of MV and its associated complications [7, 14].

Historically, weaning decisions have been the domain of physicians, with nurses primarily tasked with monitoring the patient [15]. However, neonatal nurses' continuous bedside presence enables them to observe subtle changes in the patient's condition and respond promptly during the weaning process [12].

To address the complexities of MV management, standardised weaning protocols have been introduced, resulting in earlier extubation, lower failure rates and reduced MV duration [7, 16]. The European consensus guideline highlighted the importance of managing preterm infants without MV where possible, and if ventilation is needed to minimise the time an endotracheal tube is used. On the other hand, several other respiratory support strategies, such as inhaled nitric oxide and surfactant administration, have been protocolised [17, 18]. Despite their benefits, these protocols are underutilised in NICUs compared to adult ICUs, and limited data exist on their application in neonatal populations [10].

Standardised protocols are designed to ensure consistency and improve outcomes, yet they often restrict nurses' autonomy in decision‐making [14, 19]. Autonomy, defined as the authority to act based on professional knowledge, is critical for neonatal nurses to provide individualised care [19]. In this study, autonomy refers to the neonatal nurses' ability to make ventilation decisions and implement them without direct medical supervision.

The global shortage of medical personnel, combined with nurses' consistent presence at the bedside, has expanded their roles to include traditionally physician‐driven responsibilities, often requiring them to practise beyond their formal training [20]. Competency in MV management for neonates demands specialised education, practical experience and continuous professional development [21]. Influence refers to an ability to influence decisions and affairs related to health through knowledge, effective communication and collaboration with other members of the MDT. Nurses must possess a thorough understanding of ventilator mechanisms, the clinical responses of neonates to MV and the ability to act decisively based on patient status [12, 13].

### 1.1 Justification

MV is a life‐saving intervention for neonates with respiratory distress, but its complexity demands a highly skilled and collaborative approach. While MV management involves a MDT, neonatal nurses remain the primary care providers providing continuous bedside care and making critical observations that directly impact patient outcomes. While their contributions are essential to achieving optimal patient outcomes, limited research has focused on their responsibilities and influence in this critical area. By defining their role, this study seeks to highlight their expertise and inform the development of protocols and practices that support optimal care.

## 2 Aim and Objectives

This study aimed to describe the role of neonatal nurses in MV management in NICUs.

The objectives were to:

- describe the role of neonatal nurses in key MV management practices
- explore the association between neonatal nurses' autonomy and influence on independent decision‐making to manage MV
- investigate the association between nursing contributions and influence on independent decision‐making to manage MV.

## 3 Design and Methods

### 3.1 Design

A quantitative descriptive survey design was used to collect data [22]. The design enabled the researchers to describe the role of neonatal nurses in MV management.

### 3.2 Setting

The study was conducted in the NICU, mixed neonatal and paediatric units as well as the cardiothoracic unit of two academic hospitals in Gauteng, South Africa.

### 3.3 Sample

The target population was 108 professional nurses working in the above‐mentioned institutions. The inclusion criteria were professional nurses who have worked in the NICU, mixed neonatal and paediatric unit as well as cardiothoracic unit for 6 months and longer. The cardiothoracic unit was included because it admits neonatal and paediatric patients who require cardiothoracic‐related surgeries. Census sampling method was utilised to recruit participants who met the inclusion criteria.

### 3.4 Data Collection Tools

The ‘Survey of Mechanical Ventilation and Weaning Roles and Responsibilities’ questionnaire was used to collect data [11]. This self‐administered questionnaire consists of 34 close‐ended, multiple‐choice questions and is divided into three sections. Section A consists of five items and requires the respondents' demographic data: age, academic qualification, ICU experience, current position, type of ICU and staffing ratios. Demographic variables and roles in MV were described as proportions and percentages. Section B consists of questions related to key ventilation decisions across a spectrum of MV practices (e.g., initial ventilator settings, weaning readiness, extubation readiness and weaning failure) involving nurses, either independently or collaboratively (with doctors). The last two items (autonomy and influence) are scored on a 10‐point Likert scale to determine nurses' perceived level of autonomy and influence in MV management. These were categorised as high (8–10), moderate/borderline (5–7) and low (1–4). Section C assesses the ventilation settings which are independently implemented by nurses, the presence of protocols or guidelines pertaining to MV, the use of automated weaning modes, as well as nursing education regarding MV management. The frequency of nurses independently implementing ventilation‐related decisions included titration of the fraction of inspired oxygen (FiO_2_) and the positive end expiratory pressure (PEEP). Responses were categorised as routinely (> 75%), often (51%–75%), frequently (26%–50%), seldom (25%), never (0%) and uncertain (if clarity on practice was lacking). Nurse‐to‐patient ratios were categorised for patients receiving invasive and non‐invasive ventilation, with typical options being 1:1, 1:2, 1:3 or other. Protocol availability for MV, weaning and non‐invasive ventilation was reported as yes, no and uncertain.

Each section was analysed descriptively to summarise trends, while categories (e.g., high, moderate, low) provided insights into the level of nursing involvement and perceived autonomy in MV management. These categorisations allowed for clearer understanding and presentation of results.

The ‘Survey of Mechanical Ventilation and Weaning Roles and Responsibilities’ questionnaire is a valid and reliable tool [11]. It covers a comprehensive range of topics, including identifying patient readiness for weaning and extubation, decision‐making autonomy, staffing ratios and the use of protocols. The questionnaire incorporates multiple‐choice questions and Likert‐scale items to assess constructs such as perceived autonomy and influence on patient outcomes.

The ‘Survey of Mechanical Ventilation and Weaning Roles and Responsibilities’ questionnaire demonstrates strong internal consistency, with a Cronbach's alpha of 0.85 [11]. It has been used across several European countries, including the UK, Germany, Switzerland, Italy, Greece and the Netherlands. Its design supports cross‐cultural studies by accommodating varying ICU practices.

To verify that the instrument was applicable and easily understood in the South African NICU setting, face validity was assessed by a panel of three experts. These experts were neonatal or paediatric‐trained professionals with experience in NICU practice, working as clinical facilitators, nurse educators or operational managers.

Minor modifications were made to the questionnaire based on feedback received from the experts during the face validity assessment. In Section B, the word ‘intern’ was changed to ‘medical officers’ to align with the staffing structure of the unit. Additionally, in Section C, the word ‘titration’ was changed to ‘adjustment’ as it was identified as the more commonly used term in the context of increasing or decreasing ventilator settings.

### 3.5 Data Collection

Data collection commenced after receiving ethical clearance and permissions from the study settings. The questionnaire was administered by the primary researcher in both study settings. Permission to give a brief presentation about the study at the beginning of each shift was obtained from the operational managers. All the professional nurses who met the inclusion criteria were given the participant information sheet and the questionnaire.

By completing and posting the questionnaire into the sealed box provided, the respondents were considered to have consented to participate in the study. Collection boxes for the return of the completed questionnaires were placed in a neutral area in the staff tearoom. The collection boxes were emptied twice a week.

### 3.6 Data Analysis

Statistical software (STATA version 15.0) was used for all descriptive and comparative statistical analyses. Demographic variables and roles in MV were described as proportions and percentages. For continuous variables (perceived influence and perceived autonomy scores), the Shapiro–Wilk test was computed to determine the distribution of the variable. Normally distributed data were presented as mean ± standard deviations (SD). Non‐normally distributed data were presented as medians and interquartile ranges (IQR). To determine the association between nurses' perceived autonomy and influence on independent decision‐making to manage MV, logistic regression models were used. The chi‐squared (*χ* ^2^) test was employed to assess the statistical significance of the individual predictors within the logistic regression models (where *χ* ^2^ denotes the chi‐squared statistic and df represents degrees of freedom). Odds ratios (OR) and confidence intervals (CIs) were presented at a 5% level of significance [22]. The outcome for the logistic regression model was ‘influence on independent decision‐making to MV’, which was created by running a factor analysis to reduce items (1–9) into one scale for influence on independent decision‐making to manage MV. A reliability coefficient test was computed to determine how well each item on the scale measured influence on independent decision‐making to manage MV. The outcome was then recoded into a binary outcome with two responses: ‘never/seldom’ and ‘infrequently’.

## 4 Ethical and Research Approvals

Ethical clearance from the University Human Research Ethics Committee (M180920) and permission to conduct the study were obtained from the Provincial Health Directorate, hospital management, the respective operational managers and the study participants in 2018. Informed consent was discussed during the briefing of the participants about the study, prior to issuing information letters, which also clarified that completing the questionnaire and posting it in the sealed box provided in the unit would be regarded as giving consent to take part in the study. Anonymity was ensured throughout the study, as the questionnaires did not require the respondents to include their names and information; that which was identifiable to the respondents has been represented in coded form.

## 5 Results

### 5.1 Demographics and Staffing Ratios

A total of 108 questionnaires were distributed, with 90 returned (83.3% response rate). The largest proportion of respondents (35.6%) were aged between 30 and 39 years, followed by 28.9% in the 50–59 age categories. Nearly half (47.8%) were professional nurses without an additional post‐basic qualification, and 45.6% held the role of shift leader. Regarding educational background, most (45.6%) of the respondents held an undergraduate diploma, and 25.6% had a post‐basic qualification in Critical Care Nursing and Child/Neonatal Nursing Science (13.3%), respectively. Most (54.4%) of the respondents worked in the NICU and had less than 4 years of ICU experience (40.0%). The nurse‐to‐patient ratios showed a predominant staffing ratio of 1:2 for both mechanical (56.7%) and non‐invasive ventilation (35.6%). These demographic details are summarised in Table 1.

**TABLE 1:** Demographics and staffing ratios (*n* = 90).

Category	Frequency	Percentage
Age		
20–29 years	13	14.4
30–39 years	32	35.6
40–49 years	19	21.1
50–59 years	26	28.9
> 60 years	—	—
Academic qualifications		
Undergraduate diploma (PN)	41	45.6
Undergraduate degree (PN)	14	15.6
Post‐basic child/neonatal nursing	12	13.3
Post‐basic critical care nursing	23	25.6
Years of ICU experience		
25–30 years	5	5.6
20–24 years	3	3.3
15–19 years	6	6.7
10–14 years	14	15.6
5–9 years	26	28.9
< 1–4 years	36	40.0
Position in ICU		
No response	2	2.2
Professional nurse	43	47.8
Shift leader	41	45.6
Unit manager	4	4.4
Type of ICU		
Neonatal ICU	49	54.4
Cardiothoracic ICU	18	2.0
Neonatal and Paediatric ICU	23	25.6
Staffing ratios		
What is the nurse‐to‐patient ratio for patients receiving mechanical ventilation in your ICU?		
1:1 ratio	25	27.8
1:2 ratio	51	56.7
1:3 ratio	3	3.3
Other	11	12.2
What is the nurse‐to‐patient ratio for patients receiving non‐invasive ventilation in your ICU?		
1:1 ratio	22	24.4
1:2 ratio	32	35.6
1:3 ratio	11	12.2
Other	25	27.8

*Note:* The table presents data on nurses' experience and qualifications, suggesting a potential impact on the quality of care provided in neonatal ICUs due to a possible shortage of highly experienced and specialised staff and a predominant 1:2 nurse‐to‐patient ratio for both mechanical (56.7%) and non‐invasive ventilation (35.6%).

Abbreviations: ICU: intensive care unit; PN: professional nurse.

### 5.2 Inter‐Professional Responsibility for Key Ventilation Decisions

Collaboration between nurses and doctors was the predominant approach to ventilation‐related decision‐making in this study. The majority of respondents (90.0%) reported collaborative involvement in patient evaluation and titration of ventilation settings, followed by 85.6% for assessing readiness for weaning and 80.0% for identifying weaning failure. More than half of the respondents (62.2%) indicated collaboration in ventilation setting adjustments, while 61.1% noted joint decision‐making for selecting the weaning method.

**FIGURE 1:**
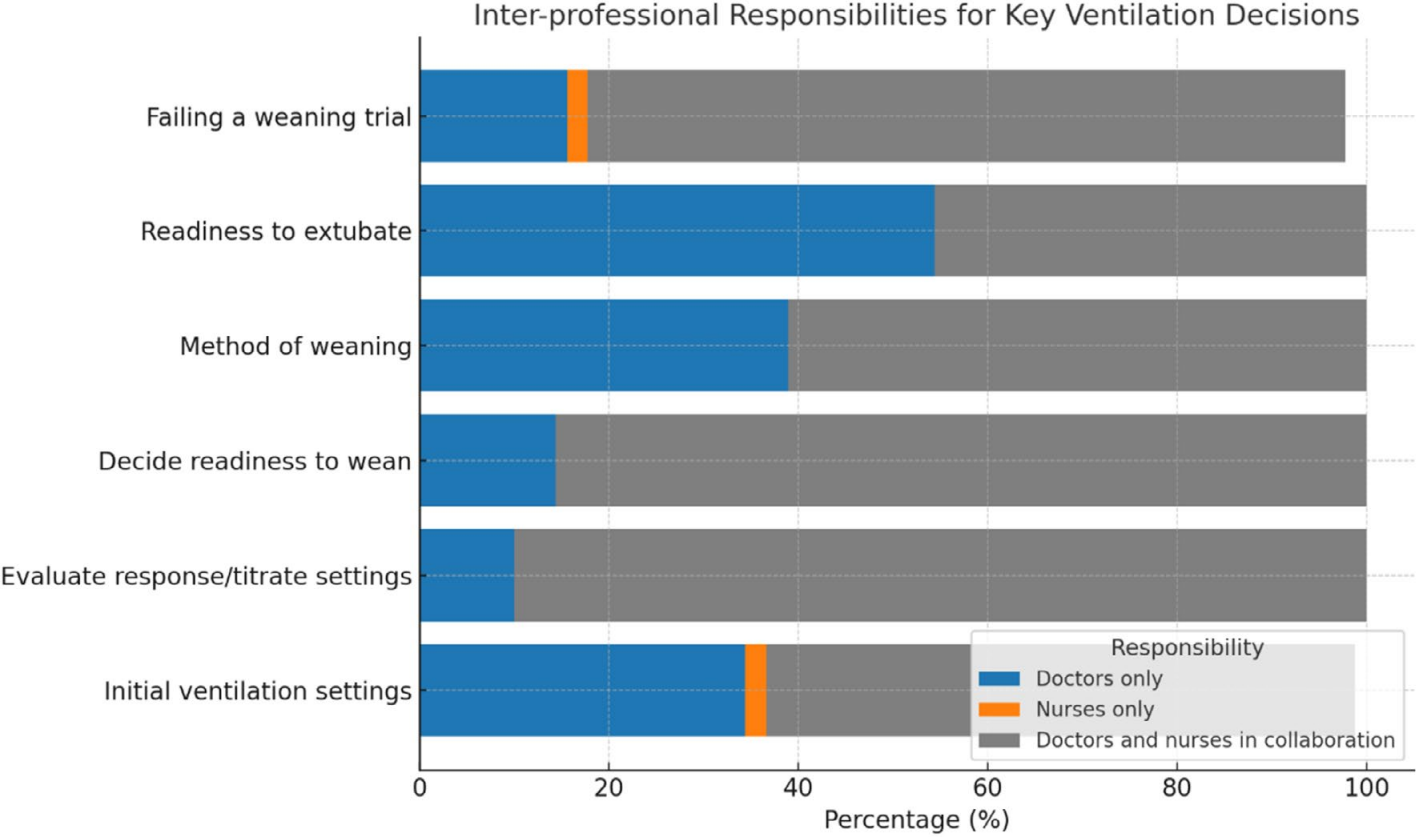
Interprofessional responsibilities for key ventilation decisions, presented as the percentage distribution of roles undertaken by doctors only, nurses only and collaboration between doctors and nurses. The data highlight the predominance of collaborative decision‐making.

However, nurses were less involved in decisions regarding extubation (45.6%), as these were primarily made by doctors (54.4%). These findings are summarised in Figure 1.

Nurses across all categories were reported to have responsibilities in various aspects of ventilation‐related decisions. Sixty‐six percent of the respondents indicated that all nurse categories were involved in decisions regarding weaning failure. A similar involvement was noted for decisions on weaning readiness (58.9%), weaning methods (57.8%) and initial ventilation settings (54.4%). Fewer respondents attributed these responsibilities exclusively to senior nurses: 43.5% for weaning readiness and 38.9% for titrating ventilation. These findings are detailed in Table 2.

**TABLE 2:** Seniority of nurses responsible for ventilation decisions.

		Senior nurses only		All nurses		Other	
		*n*	%	*n*	%	*n*	%
Q1b	Identify the seniority of nurses responsible for initial ventilation settings	28	31.1	49	54.4	13	14.4
Q2b	Identify the seniority of nurses responsible for titration of ventilation settings	35	38.9	46	51.1	9	10.0
Q3b	Identify the seniority of nurses responsible for determining weaning readiness	28	31.1	53	58.9	9	10.0
Q4b	Identify the seniority of nurses responsible for determining the method of weaning	28	31.1	52	57.8	10	11.1%
Q5b	Identify the seniority of nurses responsible for determining readiness for extubation	39	43.3	41	45.6	10	11.1
Q6b	Identify the seniority of nurses responsible for determining weaning failure	27	30.0	59	65.6	4	4.4

*Note:* The table highlights the shared responsibility for ventilation‐related decisions among nurses across all categories, with significant involvement reported in areas such as weaning failure, weaning readiness, weaning methods and initial ventilation settings.

While many key ventilation decisions are made collaboratively with doctors, in their absence, all nurses (> 50%), as opposed to only senior nurses (< 30%), were perceived responsible for key ventilation decisions. Our survey also examined the specific ventilator settings nurses reported independently titrating. For this assessment, independent titration scores were grouped into two categories: ‘frequently changed’ (meaning independent adjustments more than 50% of the time) and ‘infrequently changed’ (meaning independent adjustments less than 50% of the time). The results revealed that FiO_2_ was the most frequently independently titrated setting by nurses, while PEEP settings were the least frequently independently titrated. Table 3 provides a summary of these independent titration practices among nurse respondents.

**TABLE 3:** Type of key ventilator settings made independently by nurses.

	Frequently (> 50% of the time)		Infrequently (< 50% of the time)	
	*n*	%	*n*	%
Increase in FiO_2_	66	73.3	24	26.7
Decrease in FiO_2_	63	70.0	27	30.0
Titration of respiratory rate	25	27.8	65	72.2
Decrease in pressure support	16	17.8	74	82.2
Increase in pressure support	15	16.7	75	83.3
Change mode	14	15.6	76	84.4
Titration of inspiratory pressure	13	14.5	77	85.6
Increase in PEEP	7	7.8	83	92.2
Decrease in PEEP	5	5.6	85	94.4

*Note:* The table displays the frequency with which nurses titrate various ventilation settings, categorised as frequently changed (> 50% of the time) or infrequently changed (< 50% of the time). Oxygen (FiO_2_) was the most frequently titrated setting, while PEEP was the least frequently titrated.

Abbreviations: FiO_2_ = fraction of inspired oxygen; PEEP = positive end‐expiratory pressure.

### 5.3 Automated Modes and Protocols for Ventilation Management

The availability of automated modes is not universal in South Africa. Close to three‐quarters (76.7%) of the respondents had not worked in units that have ‘Volume A/C’ modes available. Similarly, 68.9% and 51.1% did not have ‘TCPL SIMV’ and ‘TCPL A/C’, respectively (Table 4). In this study, 36.7% of the nurses practised in ICUs in which there were protocols for ventilation management. Similarly, 37.8% of the respondents had a protocol for non‐invasive ventilation management. Of those who had protocols and guidelines, 43.3% indicated that these documents included specific guidance on the management of weaning failure and 36.7% included information for weaning. These results are displayed in Table 5.

**TABLE 4:** Use of automated modes.

Automated modes	Never/seldom		Frequently, often or routinely		Uncertain	
	*n*	%	*n*	%	*n*	%
Pressure A/C	43	47.8	43	47.8	4	4.4
Pressure SIMV	38	42.4	49	54.5	3	3.3
TCPL SIMV	62	68.9	20	22.2	8	8.9
TCPL A/C	46	51.1	34	37.8	10	11.1
Volume A/C	69	76.7	17	18.8	4	4.4
Volume SIMV	64	7.1	20	22.2	6	6.7

*Note:* The table highlights the limited availability of certain automated ventilation modes, with a majority of respondents reporting no access to Volume A/C (76.7%), TCPL SIMV (68.9%) and TCPL A/C (51.1%).

Abbreviations: A/C = assist control; SIMV = synchronised intermittent mandatory ventilation; TCPL = time‐cycled pressure limited.

**TABLE 5:** Use of protocols.

Statement	Yes		No		Uncertain	
	*n*	%	*n*	%	*n*	%
In your ICU, do you have guidelines/policy/protocol for management of mechanical ventilation?	33	36.7	39	43.3	18	20.0
In your ICU, do you have a guideline/policy/protocol for weaning from mechanical ventilation? If yes, does it contain information on management of patients failing weaning?	33	36.7	44	48.9	13	14.4
If yes, does it contain information on management of patients failing weaning?	33	43.3	39	43.3	12	13.3
In your ICU, do you have a guideline/policy/protocol for management of non‐invasive ventilation?	33	37.8	39	43.3	17	18.9

*Note:* The table highlights the reported availability of guidelines, policies or protocols for MV, weaning from MV and non‐invasive ventilation (NIV) in ICUs. A substantial proportion of nurses reported a lack of protocols for each of these respiratory support modalities.

### 5.4 Perceived Nurse Autonomy, Influence and Ventilator Education

**FIGURE 2:**
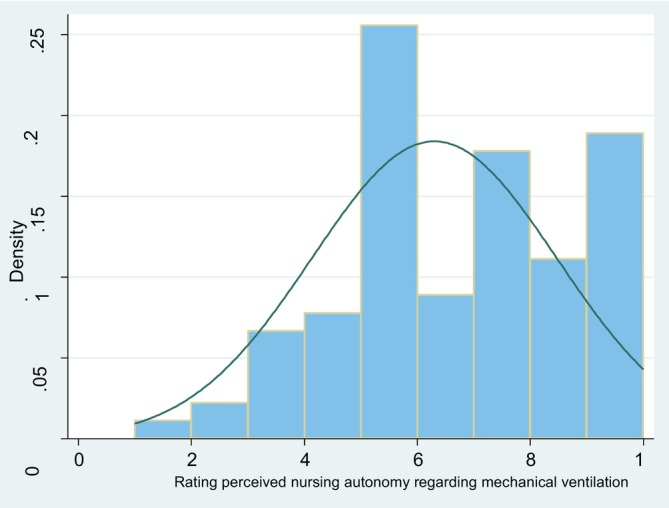
Perceived nurse autonomy in mechanical ventilation (MV) decision‐making, measured using a visual analogue scale ranging from 1 (no autonomy) to 10 (complete autonomy). The median of 6 indicates moderate perceived autonomy.

Perceived nurse autonomy on decision‐making on MV practices was assessed using a visual analogue scale (VAS). The responses ranged between 1 (no autonomy) and 10 (complete autonomy) and a median score of 6. These results are summarised in Figure 2.

To explore the association between perceived autonomy and independent decision‐making, logistic regression analysis was performed. OR and 95% CIs were used to quantify this relationship, and the *χ* ^2^ test was performed to assess statistical significance. Results showed a significant association: Higher autonomy scores increased the likelihood of independent decision‐making (OR = 1.55; 95% CI = 1.22–1.97; *χ* ^2^ (1) = 12.86; *p* < 0.001). Table 6 displays these results. Thresholds for low, borderline and high autonomy were based on the VAS. This is detailed in the instrument description.

**TABLE 6:** Association between nurses' autonomy and independent decision‐making in mechanical ventilation.

Variable	Unadjusted model				Adjusted model			
	OR (95% CI)	*χ* ^2^	df	*p*	OR (95% CI)	*χ* ^2^	df	*p*
Nursing autonomy	1.54 (1.21–1.96)	12.28	1	< 0.001^a^	1.55 (1.22–1.97)	12.86	1	< 0.001^a^
Years of experience	1.06 (0.99–1.13)	3.00	1	0.120	1.06 (0.99–1.15)	2.33	1	0.104

*Note:* The table presents the results of a logistic regression analysis, demonstrating higher perceived autonomy is a significant predictor of independent decision‐making in both models (unadjusted OR = 1.54, 95% CI: 1.21–1.96; adjusted OR = 1.55, 95% CI: 1.22–1.97; *p* < 0.001). Years of experience was not statistically significant.

Abbreviations: *χ* ^2^ = chi‐squared; CI = confidence interval; df = degrees of freedom; OR = odds ratio.

^a^ Statistically significant.

**FIGURE 3:**
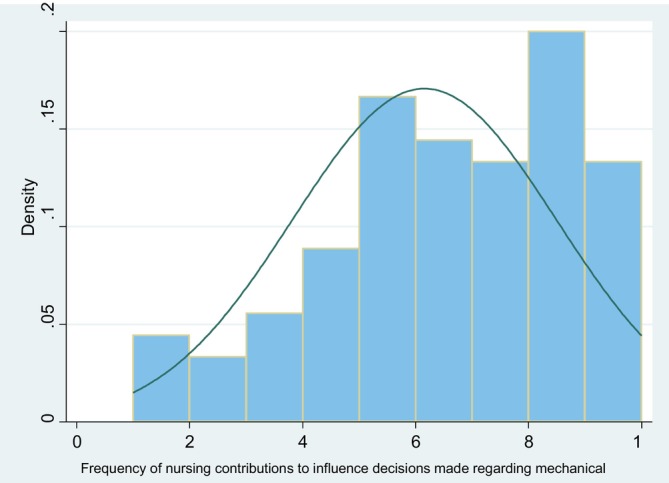
Perceived influence in mechanical ventilation (MV) decision‐making, measured using a visual analogue scale ranging from 1 (no influence) to 10 (complete influence). The median perceived influence score was 6, indicating moderate perceived influence.

Perceived nurse influence was similarly assessed via VAS, with responses ranging from 1 to 10 (median 6). Figure 3 illustrates these results.

The association between perceived influence and independent decision‐making was analysed using logistic regression. Results indicated higher influence scores significantly predicted autonomous decisions (OR = 1.86; 95% CI = 1.40–2.47; *χ* ^2^ (1) = 18.34; *p* < 0.001). This reinforces that higher perceived influence correlates with greater autonomy in MV management (Table 7).

**TABLE 7:** Association between nursing contributions and influence on independent decision‐making in mechanical ventilation.

Variable	Unadjusted model				Adjusted model			
	OR (95% CI)	*χ* ^2^	df	*p*	OR (95% CI)	*χ* ^2^	df	*p*
Nursing contributions	1.81 (1.38–2.38)	18.19	1	< 0.001^a^	1.86 (1.40–2.47)	18.34	1	< 0.001^a^
Years of experience	1.06 (0.99–1.13)	3.00	1	0.120	1.09 (0.99–1.20)	3.08	1	0.052

*Note:* The table depicts nursing contributions that were significantly associated with increased independent decision‐making in both models (unadjusted OR = 1.81, 95% CI: 1.38–2.38; adjusted OR = 1.86, 95% CI: 1.40–2.47; *p* < 0.001). Years of experience was not a significant predictor.

Abbreviations: *χ* ^2^ = chi‐squared; CI = confidence interval; df = degrees of freedom; OR = odds ratio.

^a^ Statistically significant.

Most (80.0%) of the respondents indicated that ventilation management education was not provided during orientation in the ICU, while 70.0% reported that ongoing professional development opportunities were not available. Table 8 summarises these results.

**TABLE 8:** Ventilation management education for nurses.

Statement	Yes		No		Uncertain	
	*n*	%	*n*	%	*n*	%
Do nurses receive education on ventilation during ICU orientation?	16	17.8	72	80.0	2	2.2
Are opportunities available for ICU ongoing professional development related to mechanical ventilation?	16	17.8	63	70.0	11	12.2

*Note:* The table highlights the reported lack of MV education for nurses, with a majority (80.0%) indicating no education during ICU orientation and a substantial proportion (70.0%) reporting no access to ongoing professional development in this area.

## 6 Discussion

In this study, we sought to describe the neonatal nurses' role in MV management in South African neonatal and paediatric ICUs. The findings highlighted both collaborative practices and notable challenges in MV decision‐making and education.

Our study results revealed that 28.9% of respondents had less than 10 years of ICU experience, and only 25.6% held a post‐basic qualification in Critical Care Nursing, with 13.3% specialising in Child/Neonatal Nursing Science. These findings highlight a potential shortage of highly experienced and specialised nursing staff in neonatal ICUs, which may impact the quality of care provided. Nurses with limited ICU experience and no post‐basic qualification in Child/Neonatal Nursing Science may lack advanced skills and confidence in managing MV, potentially leading to delays in critical decision‐making and increased risks for ventilated neonates. The fact that fewer than half of the respondents held post‐basic qualifications in either Critical Care or Child/Neonatal Nursing underscores a significant gap in specialist nursing capacity. This shortage can be partly explained by the phasing out of legacy specialist nurse training programmes in South Africa, which previously offered structured pathways for developing advanced clinical competencies. The discontinuation of these programmes has likely contributed to the limited availability of specialist nurses, thereby affecting the workforce's overall capacity and confidence in managing MV in NICUs. The findings of a study by Nobahar et al. [23] emphasise the importance of refining clinical decision‐making strategies for neonatal nurses, suggesting that experience and training are crucial for effective practice.

The organisation and structure of the units showed that the nurse‐to‐patient ratio was 1:2 for both invasive and non‐invasively ventilated patients. While this ratio is considered standard in many NICUs, it places considerable responsibility on nurses, particularly when managing critically ill neonates requiring MV. Less experienced nurses might feel overwhelmed, leading to burnout and errors in delivery of care to neonates. In contrast to our study findings, the results of a Norwegian study found that 92% of the ICUs had a nurse‐to‐patient ratio of 1:1 for intubated patients, and 69.0% of these ICUs had a nurse‐to‐patient ratio of 1:1 for patients receiving non‐invasive MV [24].

The respondents in our study agreed that nurses and doctors collaborated on the titration of ventilation settings. Ninety percent reported collaborative involvement in patient evaluation and titration of ventilation settings, aligning with findings from a Norwegian survey where collaboration was prevalent in determining patients' weaning readiness [24]. However, despite this collaboration, 54.4% of the nurses in our study indicated that doctors are less likely to involve them in extubation decisions. Similarly, Blackwood et al. [16] observed that while key decisions were collaborative, nurses often faced limitations in adjusting ventilator settings independently. These limitations were linked to the absence of standardised competency programmes and the infrequent use of nurse‐led weaning protocols and automated systems.

Regarding independent titrations of ventilator settings without direct medical consultation, FiO_2_ was the most frequently adjusted ventilator setting. This may be associated with the fact that changes to FiO_2_ settings are less complicated for a nurse to make than making changes to PEEP settings. This concurs with the views of the nurses in a Norwegian study [24] and an Australian study [25].

Relating to the perceived level of nursing autonomy and influence in decision‐making, findings revealed a median score of 6.0 for both. This suggests nurse respondents in this study perceived themselves to have reasonable levels of autonomy and influence in decision‐making. These results lie midway in comparison with the Norwegian survey, which stated that nurse managers perceived nurses to have a higher level of autonomy and influence (median scores of 7 and 8) as opposed to doctors, who reported a different level of nurses' autonomy and influence (*M* = 5 and 7) respectively [24].

Our results are lower than those in the studies of Rose et al. [11] and Haugdahl et al. [24] where a median score of 7.7 and 8.0 was reported for nursing influence in ventilation decisions. Similarly, in another European study [26], found nurse managers rated a median score of 7.0 for both scales. These scores may be associated with the fact that most of the respondents in our study were less experienced and were not specialist nurses. Less experienced nurses are not likely to make independent decisions without first consulting a doctor. This can delay critical decisions, such as adjusting ventilator settings or escalating care when neonates show signs of deterioration.

Nurses with a higher level of autonomy and influence scores frequently (> 50% of the time) titrated FiO_2_, respiratory rate and pressure support without medical consultation. Haugdahl et al. [24] and Rose et al. [25] also revealed that the decision to adjust FiO_2_ was the most frequently made by the nurse independently, as seen in this study.

The role of the nurses who worked in units with weaning protocols frequently (> 50% of the time) titrated the mode of ventilation, respiratory rate, inspiratory pressure, pressure support and decreased PEEP. It could be argued that the presence of protocols can enhance nurses' level of autonomy in decision‐making, as noted in the study of Blackwood et al. [27]. The minimal involvement of nurses in adjusting key ventilator settings was linked to the low prevalence of nurse‐led weaning protocols in the United Kingdom's paediatric ICUs [27]. Less experienced nurses may rely heavily on standardised protocols without adapting to the individual needs of neonates, potentially impacting personalised care.

Thirty‐six percent of the respondents in this study reported that weaning protocols were present in the NICU. Despite the lack of compelling evidence to support their use in neonates [7, 17, 28, 29] a considerable number of NICUs have adopted MV protocols [4, 10, 27].

Eighty percent of nurses reported that ventilation management education was not provided during ICU orientation, and 70.0% indicated they were not offered ongoing training on MV. It was noted from the demographic data that 43% of the respondents had an undergraduate diploma level nursing qualification. Nurses who have not worked in the ICU are likely to enter the ICU environment with limited or lack of knowledge on MV; therefore, comprehensive continuous education is crucial [11, 30].

## 7 Limitations

The limitations to our study include the following:

- The study was conducted in two academic hospitals of one province in South Africa, from a sample of 90 nurses, which may limit the generalisability of findings to other contexts.
- The majority of the respondents had limited ICU experience and no post‐basic qualification in Child/Neonatal Nursing Science and may lack advanced skills and confidence in managing MV.
- The self‐reported nature of the questionnaire responses introduces the potential for response bias.
- The inclusion of the cardiothoracic unit might have led to different perspectives on autonomy and influence; since the nurses in this unit were exposed to few neonates and paediatric patients.
- The participants in this study were nurses only; the inclusion of the doctors might have yielded different perspectives on interprofessional collaboration.

In consideration of these limitations, the findings of this study cannot be generalised unless replication of the study is carried out on a larger scale including NICUs in other public and private hospitals.

## 8 Implications and Recommendations for Practice

The findings of this study underscore critical implications for both education and clinical practice in MV management.

- The shortage of highly experienced neonatal nurses and the limited number of post‐basic qualified nurses in Critical Care Nursing and Child/Neonatal Nursing Science suggest a need for targeted training programmes.
- Incorporating structured, competency‐based educational interventions during orientation and ongoing professional development is essential to enhance nurses' confidence and expertise in MV management.
- Furthermore, the 1:2 nurse‐to‐patient ratio, while standard, places considerable demands on neonatal nurses, particularly those with less ICU experience. This highlights the need for staffing policies that consider experience levels when assigning nurses to high‐acuity neonates requiring MV.
- Expanding mentorship programmes and structured preceptorships can provide less experienced nurses with necessary guidance in decision‐making for MV.
- The study also highlights the importance of interdisciplinary collaboration. While nurses were actively involved in MV‐related decision‐making, their limited participation in extubation decisions indicates a gap in their perceived and actual autonomy.
- Strengthening nurse‐led protocols and interdisciplinary training could bridge this gap, ensuring that nurses are adequately prepared to contribute meaningfully to critical ventilation decisions.

## 9 Conclusion

This study provides valuable insights into the role of neonatal nurses in MV management, emphasising the importance of experience, education and interdisciplinary collaboration. While nurses actively participate in ventilation‐related decisions, their autonomy remains limited, particularly in extubation decisions. The findings highlight the need for targeted educational interventions, structured training programmes and standardised protocols to enhance nurse competency and confidence in MV management. The shortage of nurses with post‐basic qualifications in critical care and neonatal care nursing could be linked to the phasing out of legacy training programmes in South Africa, further emphasising the need for the development of alternative pathways to specialisation. Strengthening interdisciplinary collaboration and increasing access to ventilation management education can further empower neonatal nurses to take a more active role in critical decision‐making. Addressing these gaps will ultimately contribute to improved patient outcomes and more efficient MV management in NICUs.

## Ethics Statement

Ethical approval for this study was obtained from the University of the Witwatersrand Human Research Ethics Committee (Medical; approval number: M180920; approved in September 2018).

## Consent

The study participants were not patients.

## Conflicts of Interest

The authors declare no conflicts of interest.

## Supporting information


**Data S1:** Survey of mechanical ventilation and weaning roles and responsibilities.

## Data Availability

The data that support the findings of this study are available on request from the corresponding author. The data are not publicly available due to privacy or ethical restrictions.
