# Supplementary material for: The Role of Neonatal Nurses in Mechanical Ventilation Management
Source: Nurs Crit Care. 2025 Aug 18;30(5):e70150. doi: 10.1111/nicc.70150 (PMC12359019; doi:10.1111/nicc.70150)
Supplement: Supplementary file 1 — Data S1: Survey of mechanical ventilation and weaning roles and responsibilities. [file NICC-30-0-s001.docx]

**Survey of Mechanical Ventilation and Weaning Roles and Responsibilities**

**SECTION A**: Biographical Data

- 1. What age group do you belong to:

| 20-29 years |  |
| --- | --- |
| 30-39 years |  |
| 40-49 years |  |
| 50-60 years |  |
| >60 years |  |

- 1. Please list your nursing academic qualifications
  2. State the year you qualified (years of experience) as an Intensive Care nurse

|  |
| --- |

- 1. Indicate your position in the ICU by ticking one of the following:

| Professional nurse | Shift leader | Unit manager |
| --- | --- | --- |

- 1. Indicate your clinical area by ticking one of the following:

| Neonatal ICU |  |
| --- | --- |
| Paediatric ICU |  |
| Cardiothoracic ICU |  |

**SECTION B:** KEY VENTILATION DECISIONS, STAFFING RATIOS, AUTONOMY AND INFLUENCE

1. Who determines the initial selection of ventilator settings?

| a | Doctors only |  |
| --- | --- | --- |
| b | Nurses only |  |
| c | Doctors and nurses in collaboration |  |
| d | Other |  |

If other, please specify ________________________________________________________________________________________________________________________________________

- 1. Identify the seniority of doctors responsible for initial selection of ventilator settings

| a | Consultants only |  |
| --- | --- | --- |
| b | Registrars and above |  |
| c | Interns and above |  |
| d | Other |  |

If other, please specify

________________________________________________________________________________________________________________________________________

- 1. If applicable, the seniority of nurses responsible for initial selection of ventilator settings

| a | Senior nurses only (e.g. clinical nurse specialists, nurse managers, educators |  |
| --- | --- | --- |
| b | All nursing staff (once orientated to the ICU environment) |  |
| c | Other |  |

If other, please specify

________________________________________________________________________________________________________________________________________

1. Who evaluates the patient’s response to mechanical ventilation and titrates settings if required?

| a | Doctors only |  |
| --- | --- | --- |
| b | Nurses only |  |
| c | Doctors and nurses in collaboration |  |
| d | Other |  |

If other, please specify

________________________________________________________________________________________________________________________________________

- 1. Identify the seniority of doctors responsible for titration of ventilator settings

| a | Consultants only |  |
| --- | --- | --- |
| b | Registrars and above |  |
| c | Interns and above |  |
| d | Other |  |

If other, please specify ________________________________________________________________________________________________________________________________________

- 1. If applicable, identity the seniority of nurses responsible for titration of ventilator settings

| a | Senior nurses only (e.g. clinical nurse specialists, nurse managers, educators) |  |
| --- | --- | --- |
| b | All nursing staff (once orientated to the ICU environment) |  |
| c | Other |  |

If other, please specify ________________________________________________________________________________________________________________________________________

1. Who decides when a patient is ready to wean?

| a | Doctors only |  |
| --- | --- | --- |
| b | Nurses only |  |
| c | Doctors and nurses in collaboration |  |
| d | Other |  |

If other, please specify

________________________________________________________________________________________________________________________________________

- 1. Identify the seniority of doctors responsible for determining weaning readiness

| a | Consultants only |  |
| --- | --- | --- |
| b | Registrars and above |  |
| c | Interns and above |  |
| d | Other |  |

If other, please specify

________________________________________________________________________________________________________________________________________

- 1. If applicable, identify the seniority of nurses responsible for determining weaning readiness

| a | Senior nurses only (e.g. clinical nurse specialists, nurse managers, educators) |  |
| --- | --- | --- |
| b | All nursing staff (once orientated to the ICU environment) |  |
| c | Other |  |

If other, please specify

________________________________________________________________________________________________________________________________________

1. Who decides the method of weaning from mechanical ventilation?

| a | Doctors only |  |
| --- | --- | --- |
| b | Nurses only |  |
| c | Doctors and nurses in collaboration |  |
| d | Other |  |

If other, please specify

________________________________________________________________________________________________________________________________________

- 1. Identify the seniority of doctors responsible for determining the method of weaning

| a | Consultants only |  |
| --- | --- | --- |
| b | Registrars and above |  |
| c | Interns and above |  |
| d | Other |  |

If other, please specify

_______________________________________________________________________________________________________________________________________

- 1. If applicable, identify the seniority of nurses responsible for determining the method of weaning

| a | Senior nurses only (e.g. clinical nurse specialists, nurse managers, educators) |  |
| --- | --- | --- |
| b | All nursing staff (once orientated to the ICU environment) |  |
| c | Other |  |

If other, please specify

________________________________________________________________________________________________________________________________________

1. Who decides when a patient is ready to extubate?

| a | Doctors only |  |
| --- | --- | --- |
| b | Nurses only |  |
| c | Doctors and nurses in collaboration |  |
| d | Other |  |

If other, please specify

________________________________________________________________________________________________________________________________________

- 1. Identify the seniority of doctors responsible for determining readiness for extubation.

| a | Consultants only |  |
| --- | --- | --- |
| b | Registrars and above |  |
| c | Interns and above |  |
| d | Other |  |

If other, please specify

________________________________________________________________________________________________________________________________________

- 1. If applicable, identify the seniority of nurses responsible for determining readiness for extubation.

| a | Senior nurses only (e.g. clinical nurse specialists, nurse managers, educators) |  |
| --- | --- | --- |
| b | All nursing staff (once orientated to the ICU environment) |  |
| c | Other |  |

If other, please specify

____________________________________________________________________________________________________________________________________________________

1. Who decides when a patient is failing a weaning trial?

| a | Doctors only |  |
| --- | --- | --- |
| b | Nurses only |  |
| c | Doctors and nurses in collaboration |  |
| d | Other |  |

If other, please specify

____________________________________________________________________________________________________________________________________________________

- 1. Identify the seniority of doctors responsible for determining weaning failure

| a | Consultants only |  |
| --- | --- | --- |
| b | Registrars and above |  |
| c | Interns and above |  |
| d | Other |  |

If other, please specify

________________________________________________________________________________________________________________________________________

- 1. If applicable, identify the seniority of nurses responsible for determining weaning failure

| a | Senior nurses only (e.g. clinical nurse specialists, nurse managers, educators) |  |
| --- | --- | --- |
| b | All nursing staff (once orientated to the ICU environment) |  |
| d | Other |  |

If other, please specify

________________________________________________________________________________________________________________________________________

1. What is the nurse-to-patient ratio for patients receiving mechanical ventilation in your ICU?

| a | 1:1 ratio |  |
| --- | --- | --- |
| b | 1:2 ratio |  |
| c | 1:3 ratio |  |
| d | Other |  |

If other, please specify

________________________________________________________________________________________________________________________________________

1. What is the nurse-to-patient ratio for patients receiving non-invasive ventilation in your ICU?

| a | 1:1 ratio |  |
| --- | --- | --- |
| b | 1:2 ratio |  |
| c | 1:3 ratio |  |
| d | Other |  |

If other, please specify

________________________________________________________________________________________________________________________________________

1. How would you rate nursing autonomy with regard to mechanical ventilation practices? Please circle the number on the scale below (10 represents the highest level of autonomy and 1 represents the lowest level).

| 1 | 2 | 3 | 4 | 5 | 6 | 7 | 8 | 9 | 10 |
| --- | --- | --- | --- | --- | --- | --- | --- | --- | --- |

1. How often do nursing contributions influence decisions made regarding mechanical ventilation? Please circle the number on the scale below (10 represents very often and 1 represents seldom).

| 1 | 2 | 3 | 4 | 5 | 6 | 7 | 8 | 9 | 10 |
| --- | --- | --- | --- | --- | --- | --- | --- | --- | --- |

**SECTION C:** INDEPENDENT VENTILATION DECISIONS, PROTOCOLS, AUTOMATED WEANING AND NURSE EDUCATION

1. How often do nurses make and implement the following decisions independently? (without direct consultation with a doctor):

|  | Never (0%) | Seldom (25%) | Frequently (26-50%) | Often  (51-75%) | Routinely (>75%) | Uncertain |
| --- | --- | --- | --- | --- | --- | --- |
| 1.1 Change of mode |  |  |  |  |  |  |
| 1.2Titration of respiratory rate |  |  |  |  |  |  |
| 1.3 Titration of inspiratory pressure |  |  |  |  |  |  |
| 1.4 Increase of pressure support |  |  |  |  |  |  |
| 1.5 Decrease of pressure support |  |  |  |  |  |  |
| 1.6 Increase of PEEP |  |  |  |  |  |  |
| 1.7 Decrease of PEEP |  |  |  |  |  |  |
| 1.8 Increase of FiO2 |  |  |  |  |  |  |
| 1.9 Decrease of FiO2 |  |  |  |  |  |  |

1. In your ICU, do you have guidelines/policy/protocol for management of mechanical ventilation?

| Yes |  | No |  | Uncertain |  |
| --- | --- | --- | --- | --- | --- |

1. In your ICU, do you have a guideline/policy/protocol for weaning from mechanical ventilation?

| Yes |  | No |  | Uncertain |  |
| --- | --- | --- | --- | --- | --- |

3.1 If yes, does it contain information on management of patients failing weaning?

| Yes |  | No |  | Uncertain |  |
| --- | --- | --- | --- | --- | --- |

1. In your ICU, do you have a guideline/policy/protocol for management of non- invasive ventilation?

| Yes |  | No |  | Uncertain |  |
| --- | --- | --- | --- | --- | --- |

1. Are there any automated weaning modes used in your ICU? Please add any other modes in empty boxes provided below (if applicable)

|  | Never (0%) | Seldom (25%) | Frequently (26-50%) | Often  (51-75%) | Routinely (>75%) | Uncertain |
| --- | --- | --- | --- | --- | --- | --- |
| 5.1 Pressure A/C |  |  |  |  |  |  |
| 5.2 Pressure SIMV |  |  |  |  |  |  |
| 5.3 TCPL SIMV |  |  |  |  |  |  |
| 5.4 TCPL A/C |  |  |  |  |  |  |
| 5.5 Volume A/C |  |  |  |  |  |  |
| 5.6 Volume SIMV |  |  |  |  |  |  |

1. Do nurses receive education on ventilation during ICU orientation?

| Yes |  | No |  | Uncertain |  |
| --- | --- | --- | --- | --- | --- |

6.1 If YES please describe (optional)

|  |
| --- |

1. Are opportunities available in your ICU for on-going professional development related to mechanical ventilation?

| Yes |  | No |  | Uncertain |  |
| --- | --- | --- | --- | --- | --- |

7.1 If YES please describe (optional)

|  |
| --- |

**Adapted Survey of Mechanical Ventilation and Weaning Roles and Responsibilities (Rose et al., 2008).**
